# Deleterious Variation in BR Serine/Threonine Kinase 2 Classified a Subtype of Autism

**DOI:** 10.3389/fnmol.2022.904935

**Published:** 2022-06-10

**Authors:** Jingxin Deng, Yi Wang, Meixin Hu, Jia Lin, Qiang Li, Chunxue Liu, Xiu Xu

**Affiliations:** ^1^Division of Child Health Care, National Children’ Medical Center, Children’s Hospital of Fudan University, Shanghai, China; ^2^Shanghai Key Laboratory of Birth Defect Prevention and Control, NHC Key Laboratory of Neonatal Diseases, Translational Medical Center for Development and Disease, National Children’s Medical Center, Institute of Pediatrics, Children’s Hospital of Fudan University, Shanghai, China

**Keywords:** *BRSK2* gene, autism spectrum disorder, zebrafish, neurodevelopment, social preference, animal model

## Abstract

Recently, deleterious variants in the BR serine/threonine kinase 2 (*BRSK2*) gene have been reported in patients with autism spectrum disorder (ASD), suggesting that *BRSK2* is a new high-confidence ASD risk gene, which presents an opportunity to understand the underlying neuropathological mechanisms of ASD. In this study, we performed clinical and neurobehavioral evaluations of a proband with a *de novo* non-sense variant in *BRSK2* (p.R222X) with other reported *BRSK2* mutant patients. To validate *BRSK2* as an ASD risk gene, we generated a novel *brsk2b*-deficient zebrafish line through CRISPR/Cas9 and characterized its morphological and neurobehavioral features as well as performed molecular analysis of neurogenesis-related markers. The proband displayed typical ASD behaviors and language and motor delay, which were similar to other published *BRSK2* mutant patients. Morphologically, *brsk2b*^–/–^ larvae exhibited a higher embryonic mortality and rate of pericardium edema, severe developmental delay, and depigmentation as well as growth retardation in the early developmental stage. Behaviorally, *brsk2b^–/–^* zebrafish displayed significantly decreased activity in open field tests and enhanced anxiety levels in light/dark tests and thigmotaxis analysis. Specifically, *brsk2b^–/–^* zebrafish showed a prominent reduction of social interaction with peers and disrupted social cohesion among homogeneous groups. Molecularly, the mRNA expression levels of *homer1b* (a postsynaptic density scaffolding protein), and *mbpa, mpz*, and *plp1b* (molecular markers of oligodendrocytes and myelination) were increased in the brain tissues of adult *brsk2b^–/–^* zebrafish, while the expression level of *isl1a*, a marker of motor neurons, was decreased. Taken together, for the first time, we established a novel *brsk2b*-deficient zebrafish model that showed prominent ASD-like behaviors. In addition, the disturbed mRNA expression levels of neurogenesis-related markers implied that the processes of postsynaptic signaling as well as oligodendrocytes and myelination may be involved. This discovery may suggest a path for further research to identify the underlying neuropathological mechanisms between *BRSK2* and ASD.

## Introduction

Autism spectrum disorder (ASD) is a heterogeneous neurodevelopmental disorder manifesting in early development that is characterized by persistent deficits in social interaction and restricted, repetitive, or unusual sensory–motor behaviors (Lord et al., 2020). Dozens of cohort studies have elucidated that the heritability of ASD is estimated to range from 60 to 90% (Tick et al., 2016; Bai et al., 2019; Hansen et al., 2019; Castelbaum et al., 2020). Sibling studies have indicated that the risk of ASD occurrence of a subsequent child following an older child with ASD is 8.4-fold higher compared to the risk in unaffected families (Hansen et al., 2019), supporting a strong genetic contribution to ASD pathogenesis. So far, over 100 genetic loci and rare genetic variants have been identified to contribute to ASD (Ronemus et al., 2014; Satterstrom et al., 2020). Identifying additional risk genes associated with ASD will provide pathological insight into our understanding of ASD etiology.

Recently, a novel gene, BR serine/threonine kinase 2 (*BRSK2*, NC_000011.10), was identified as a high-confidence ASD risk gene (Feliciano et al., 2019). A study reported nine patients with rare, heterozygous variants in *BRSK2* from cohorts including 3,429 probands with developmental delay/intellectual disability (DD/ID). Six of the variants were loss-of-function and three were predicted to be damaging missense variants. In particular, 78% (7/9) patients were diagnosed with ASD (Hiatt et al., 2019). Another study (Feliciano et al., 2019) that assessed whole-exome sequencing and genotyping data of 457 autism families provided statistical support for 26 ASD risk genes at a false discovery rate of 0.1 in the SPARK cohort of ASD families. Importantly, among these genes, *BRSK2* is the only one with multiple *de novo*, likely gene-disrupting variants that reached genome-wide significance supporting *BRSK2* as a novel risk gene for ASD. Also known as *SAD1* or *SADA, BRSK2* is located at 11p15.5 and is exclusively expressed in the human central nervous system and pancreas. Along with its homolog BRSK1 (also known as SAD2 or SAD-B, NP_115806.1), the BRSK2 (NP_001243556.1) protein belongs to the AMPK-related family of protein kinases and plays a role in axon specification and arborization by regulating microtubule dynamics through the phosphorylation of Tau after being phosphorylated by LKB1 (Kishi et al., 2005; Barnes et al., 2007). In addition, BRSK kinases have also been demonstrated to localize to synapses and regulate their maturation in the peripheral and central nervous systems (Lilley et al., 2014).

Previous rodent models have been applied to study the molecular mechanisms underlying the neurodevelopmental effects of *BRSK2* and these models displayed some of the phenotypes seen in patients. *Brsk1/Brsk2* null mutant mice displayed neurodevelopmental disruptions including severe dyskinesia and lethality within 2 h after birth as well as a morphologically aberrant thin cortex and disturbed specification of axons and dendrites (Kishi et al., 2005; Dhumale et al., 2018). Since the *Brsk1/Brsk2* mutant mice died perinatally, several conditional knockout mice were also generated. The BRSK kinases were both deleted from motor neurons by embryonic day 13.5 in *Brsk^*Isl1*–*cre*^* mice and 15.5 in *Brsk^*ChAT*–*cre*^* mice, respectively. The former died within 24 h after birth, and the latter survived until adulthood but exhibited mild postural tremors. However, *Brsk2*-single mutant mice were observed to have affected neuronal migration and died several days after birth, which was not replicated in *Brsk1* mutant mice, indicating that *BRSK2*, rather than *BRSK1*, is required for cortical development (Nakanishi et al., 2019). Another *Brsk2*-single knockout mouse developed normally without gross abnormalities, but they exhibited growth retardation and hypoinsulinemia, suggesting impaired islet β-cell function (Nie et al., 2013). Nonetheless, neurobehavioral analyses were insufficient to further study phenotype-genotype correlations when BRSK2 dysfunction was generated in these rodent models.

In conclusion, although growing evidence indicates that BRSK2 plays an important role in neurodevelopment, and mutations in the *BRSK2* gene associate with ASD, none of these studies provided direct evidence to illustrate that BRSK2 dysfunction is relevant to ASD on the basis of ASD animal models. About 60% of human ASD risk genes have orthologs in the zebrafish genome (Meshalkina et al., 2018), and the brain regions of the “social decision-making network” remain largely conserved between mammals and zebrafish (Geng and Peterson, 2019). Thus, zebrafish can be used to model the physiological functions of the human brain and have become a valuable model organism for studying social neurobiology and developmental diseases (Sakai et al., 2018; Rea and Van Raay, 2020).

In this study, we investigated the phenotype-genotype correlations of *BRSK2* mutant patients, and for the first time, we modeled a novel *brsk2*-deficient zebrafish line to analyze the developmental characteristics and neurobehavioral features relevant to ASD.

## Materials and Methods

### Laboratory Animals and Feeding Conditions

The wild-type zebrafish of TU strains were housed in the Institute of Zebrafish, Children’s Hospital of Fudan University. Both larval and adult zebrafish were maintained in standard laboratory conditions: a circulating water system at 28.5°C constant temperature and exposed to a 14-h-light/10-h-dark circadian cycle. All animal experiments were conducted in accordance with the Guiding Principles for the Care and Use of Laboratory Animals and approved by the institutional animal care committee of Children’s Hospital of Fudan University.

### Phylogenetic Tree and Comparative Genomics Analysis of BR Serine/Threonine Kinase 2 Gene

**FIGURE 1:**
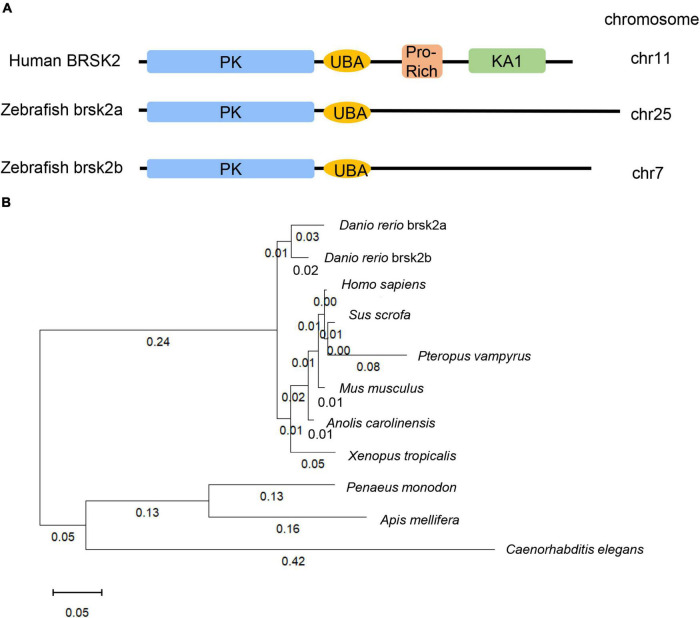
3dpf and brain tissues of Conserved and homologous analysis of BRSK2. **(A)** Comparisons of protein structure and conserved domain between human BRSK2 and zebrafish brsk2a, brsk2b. The corresponding gene *BRSK2* (NC_000011.10) locates at chr 11, *brsk2a* (NC_007136.7) locates at chr 25, *brsk2b* (NC_007118.7) locates at chr 7. **(B)** Phylogenetic analysis of BRSK2 in 10 species. The numbers on the phylogenetic tree represent evolutionary relationships, with larger numbers indicating greater genetic differences. *Homo sapiens*: NP_001243556.1, *Sus scrofa*: XP_020938244.1, *Pteropus vampyru*s: XP_023381426.1, *Mus musclulus*: NP_001009929.1, *Anolis carolinensis*: XP_016848950.1, *Danio rerio* brsk2a: XP_017209740.1, *Danio rerio* brsk2b: XP_009301703.1, *Xenopus tropicalis*: XP_004913521.1, *Penaeus monodon*: XP_037797337.1, *Apis mellifera*: XP_006557814.2, *Caenorhabditis elegans*: NP_001076761.1.

The human *BRSK2* gene duplicates into two orthologs, *brsk2a* (NC_007136.7) and *brsk2b* (NC_007118.7) in zebrafish. The nucleotide sequences, amino acid sequences and conserved domains of human BRSK2 (NP_001243556.1, 736aa), zebrafish brsk2a (XP_017209740.1, 873aa) and brsk2b (XP_009301703.1, 827aa) were obtained in the national center for biotechnology information (NCBI) gene database^1^ and were aligned for conservation and homology using the DNAMAN software (version 9.0.1.116). Sequences of BRSK2 proteins in ten species were identified using the NCBI blast program and confirmed by the best reciprocal blast (*Homo sapiens*: NP_001243556.1, *Sus scrofa*: XP_020938244.1, *Pteropus vampyrus*: XP_023381426.1, *Mus musclulus*: NP_001009929.1, *Anolis carolinensis*: XP_016848950.1, *Danio rerio* brsk2a: XP_017209740.1, *Danio rerio* brsk2b: XP_009301703.1, *Xenopus tropicalis*: XP_004913521.1, *Penaeus monodon*: XP_037797337.1, *Apis mellifera*: XP_006557814.2, *Caenorhabditis elegans*: NP_001076761.1, Figure 1B). Multiple alignments were performed and the phylogenetic tree of ten species were further conducted using MEGA 11 software by the neighbor-joining method.

### Generation of *brsk2b* Mutant Zebrafish

**FIGURE 2:**
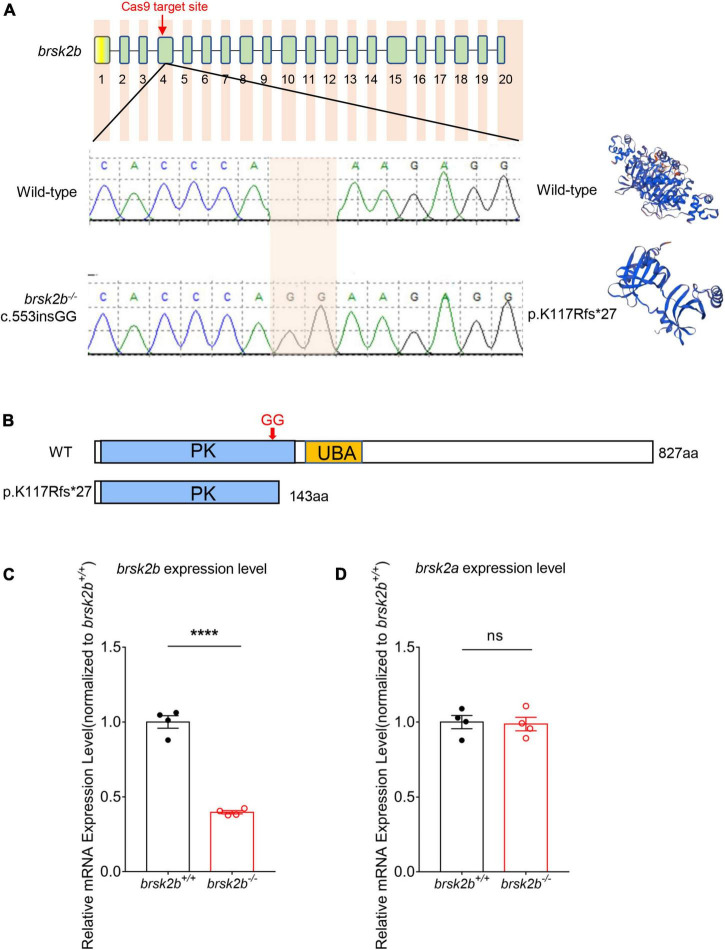
Generation of *brsk2b* mutant zebrafish. **(A)** Sanger sequencing and computational modeling of wild-type and mutant *brsk2b* in zebrafish. **(B)** The protein structures of brsk2b before and after variation by CRISPR/Cas9. **(C)** RT-qPCR showed a decrease in *brsk2b* mRNA expression in *brsk2b* mutant zebrafish (*n* = 4 for each genotype, *p* < 0.0001). **(D)** RT-qPCR showed no significant difference in *brsk2a* mRNA expression level between wild-type and *brsk2b* mutant zebrafish (*n* = 4 for each genotype, *p* = 0.847). Groups were statistically compared using an unpaired Student’s *t*-test. Data are shown as mean ± SEM, ****p < 0.001.

The *brsk2b* mutant zebrafish were generated using a CRISPR/cas9 system as previously reported (Albadri et al., 2017). The site-specific guide RNA (gRNA) of *brsk2b* was designed to target exon 4 (target site: 5′-GGGCAGGTTAACACCCAAAG-3′) (Figure 2A and Supplementary Table 1) and synthesized by *in vitro* transcription (MAXIscript™ T7 kit AM1314M, Invitrogen). 600 pg cas9 Nuclease (EnGen™ spy cas9 NLS #M0646, New England Biolabs) and 150 pg gRNA were mixed together and microinjected into one-cell stage of fertilized wild-type zebrafish embryos (F0). Injected embryos were allowed to develop to 72 h post fertilization (hpf), and then 12–20 injected embryos were randomly selected to extract genomic DNA to screen for mutations by PCR amplification and subsequent sanger sequencing. The genotyping PCR amplification conditions were as follows: 95°C, 4 min; 35 cycles of 95°C, 30 s; 58°C, 30 s; 72°C, 45 s; 72°C, 7 min (PCR primers are listed in Supplementary Table 1). F0 adult zebrafish were genotyped and crossbred with wild-type zebrafish for at least two generations to select F2 *brsk2b* heterozygous zebrafish, and then F2 fish were in-crossed to obtain homozygous mutants. The SWISS-MODEL tool^2^ was used to model and visualize the mutant and wild-type protein structures.

### Tg (*HuC*: RFP); *brsk2b*^–/–^ Transgenic Zebrafish Maintenance and Imaging

The Tg (*HuC*: RFP) transgenic line was kindly provided by Dr. Xu Wang (Fudan University), which was generated as previously described (Liu et al., 2018). We crossed *brsk2b* homozygous mutants with Tg (*HuC*: RFP) line to obtain Tg (*HuC*: RFP); *brsk2b^–/–^* transgenic zebrafish for imaging experiments. Larvae were anesthetized with tricaine at a concentration of 40 mg/L and then viewed on a Leica M205 FA fluorescence microscope at 1, 2, 3 days post fertilization (dpf). In order to maintain the comparative intensities of the fluorescence and not to introduce bias, we used the same settings (Exposure: 6 s, Saturation: 0.9, Gain: 1X, Gamma: 0.8) for all controls and Tg (*HuC*: RFP); *brsk2b^–/–^* transgenic animals per experiment. Images were taken with no auto-correction to prevent inconsistencies in fluorescence intensity and then were processed using ImageJ software.

### RNA Extraction, Reverse Transcription and RT-qPCR

Total RNA was extracted from zebrafish embryos at 1,2,3 dpf and brains at 5,7,10 dpf and 1,3,4 months post fertilization (mpf) using MiniBEST Universal RNA Extraction kit (No.9767, Takara, Japan), according to the manufacturer’s protocol. Each condition included 3–4 samples consisting of 20–30 embryos/larvae or 3–4 adult brains per sample for RNA extraction and experiments were conducted in triplicate. Reverse transcription was performed with a PrimeScript™ superscript RT Reagent Kit (RR036Q, Takara, Japan), following the manufacturer’s instruction. RT-qPCR was performed using a LightCycler^®^ 480 apparatus (Roche, Germany) and TB Green Premix Ex Taq II (Tli RNaseH Plus) (RR820A, Takara, Japan) to test the mRNA expression levels of *brsk2a, brsk2b, homer1b, nrgna, isl1a, sox2, neurog1, olig1, olig2, sox10, mpba, mpz, plp1b*, according to the manufacturers’ protocol. β*-actin* was chosen as an internal control to normalize the expression levels of target genes by using the delta CT method. The primer sequences for RT-qPCR are listed in Supplementary Table 1.

### Morphological Characteristics of *brsk2b* Mutant Zebrafish

**FIGURE 3:**
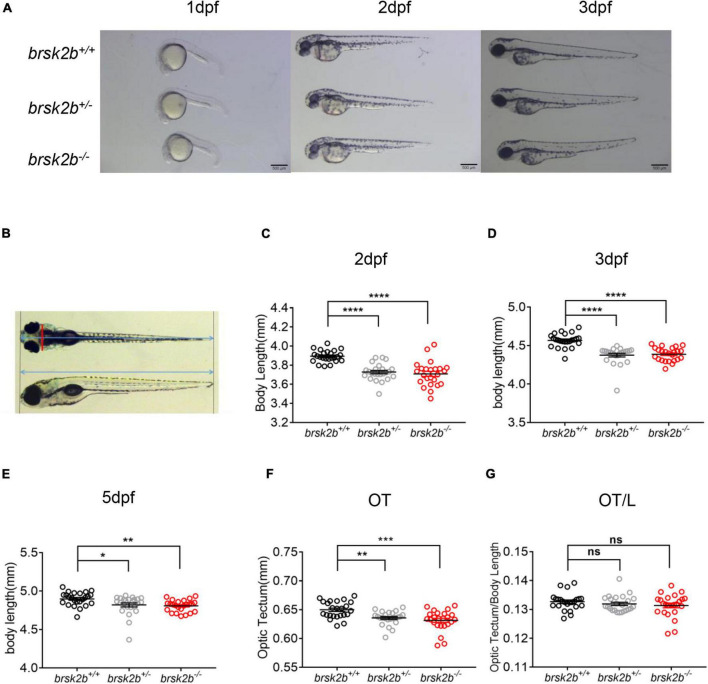
*brsk2b*-deficient zebrafish displayed developmental delay. **(A)** Lateral images of *brsk2b*^+/+^, *brsk2b^±^, brsk2b^–/–^* larvae at 1, 2, 3 dpf. Scale bars 500 μm. **(B)** The optic tectum (OT) and body length (L) were measured from lateral and dorsal images using ImageJ. The red line represents OT, and the blue lines represent body length. **(C–E)** The body lengths of *brsk2b^–/–^* larvae at 2 dpf **(C)**, 3 dpf **(D)**, 5 dpf **(E)** were significantly shorter than that of *brsk2b*^+/+^ larvae (*n* = 24 for each genotype, *p* = 0.0001, 0.0001, and 0.0043, respectively). **(F)** The values of OT of *brsk2b^–/–^* larvae at 5 dpf were smaller compared to that of *brsk2b*^+/+^ larvae (*n* = 24 for each genotype, *p* = 0.0001). **(G)** The ratio of OT/L in three genotypes didn’t show significant difference at 5 dpf (*brsk2b*^+/+^: *brsk2b*^±^: *brsk2b^–/–^* = 24: 23: 22, *p* = 0.385). Groups were compared using a one-way ANOVA followed by a Dunnett’s multiple comparisons test. Data are shown as mean ± SEM, *p < 0.05, **p < 0.01, ***p < 0.001, ****p < 0.0001.

To measure the morphological characteristics of *brsk2b* mutant zebrafish, the mortality and rate of pericardium edema, severe developmental delay and depigmentation were calculated at 1dpf, 2dpf, and 3dpf. In addition, the larvae were taken pictures at 1dpf, 2dpf, 3dpf, and 5dpf laterally or dorsally on a stereo light microscope (Leica M205 FA, United States) after anesthetized with tricaine at a concentration of 40 mg/L (Figure 3A). Then the body length was measured as the distance between the farthest most left and right of the larvae (Figure 3B). Measurements of optic tectum (OT) were also performed as the brain width directly behind the eyes in the dorsal view following previously study (Colón-Rodríguez et al., 2020; Figure 3B). All the measurements were conducted using ImageJ software.

### Larval Locomotor Activity and Light/Dark Tests

**FIGURE 4:**
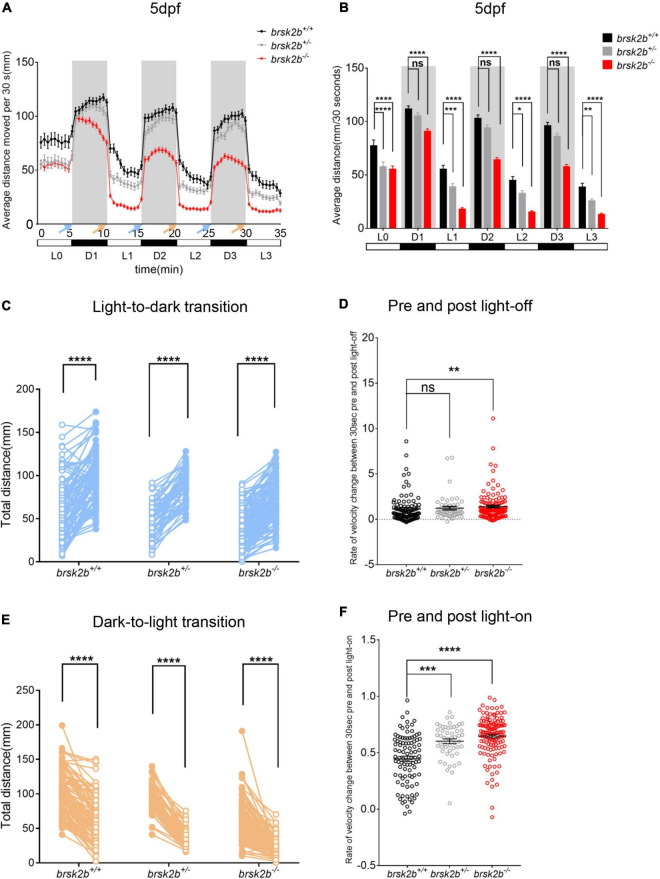
The light/dark test of *brsk2b*^+/+^, *brsk2b*^±^ and *brsk2b^–/–^* larvae at 5 dpf. **(A,B)** The activity of *brsk2b^–/–^* larvae was significantly reduced in both light and dark phases compared to *brsk2b*^±^ and *brsk2b*^+/+^ fish. The activity was analyzed for the latter 5 min of light period (L0) and three 5-min of dark/light cycles (D1, L1, D2, L2, D3, L3). Blue arrows indicate light-to-dark transition and orange arrows indicate dark-to-light transition (*brsk2b*^+/+^: *brsk2b*^±^: *brsk2b^–/–^* = 89: 52: 128). Data are presented as mean ± SEM. Within groups were compared using one-way ANOVA and Dunnett’s multiple comparisons tests. **(C)** Paired dot plots compared average swimming distances per larva of the three light conversions in the 30 s before and after the three light-to-dark conversions (*brsk2b*^+/+^: *brsk2b*^±^: *brsk2b^–/–^* = 89: 52: 128). Within genotype comparisons were conducted by paired *t*-tests. **(D)** Box plots compared average rate of velocity change in the 30 s before and after the lights-off. Boxes denote the median, 1st and 3rd quartile, while whiskers represent the minimum and maximum values. Groups were statistically compared using Kruskal–Wallis ANOVA. **(E)** Paired dot plots compared average swimming distances per larva of the three light conversions in the 30 s before and after the three dark-to-light conversions (*brsk2b*^+/+^: *brsk2b*^±^: *brsk2b^–/–^* = 89: 52: 128). Within genotype comparisons were conducted by paired *t*-tests. **(F)** Box plots compared average rate of velocity change in the 30 s before and after the lights-on. Boxes denote the median, 1st and 3rd quartile, while whiskers represent the minimum and maximum values. Groups were statistically compared using Kruskal–Wallis ANOVA. *p < 0.05, **p < 0.01, ***p < 0.001, ****p < 0.0001.

The locomotor activity of larval zebrafish at 5 dpf was analyzed by a ViewPoint setup combined with an automated computer recording system equipped with VideoTrack software. The videos were captured with a Point Gray black-and-white camera and recorded for 45 min at 25 fps. The detection threshold was set to 25. Every larva was habituated separately in 24-well plates for 15 min in the experimental chamber before video acquisition. After the former 15-min light period of general locomotor activity, the larvae experienced three dark/light cycles (5 min of dark and 5 min of light per cycle, indicated as D1, L1, D2, L2, D3, L3 in Figures 4A,B, respectively) during the latter 30 min to detect activities under a series of intermittent light stimulation. The light intensity was 100 lx in the light period and 0 lx in the dark period. The frame rate of the video was set to 25/s. The time and distance of larval activity were recorded every 30 s from transformational visual route of every larval trajectory by Zebralab software. For general activity analysis, locomotor activity was quantified as average distance moved per 30 s during the former 15-min light period. For visual motor response triggered by illumination conversion, the activity change in light/dark period was analyzed as velocity change between 30 s before and after the light was turned off or turned on.

### Open Field Tests, Stereotype, and Thigmotaxis Analysis

The locomotor activity of adult zebrafish was tested at 4 mpf in an open field paradigm. Videos were captured in a 30 × 30 × 30 cm opaque tank filled with system water using a suspended camera right above. Each male zebrafish was habituated in the tank for 5 min before the 30-min test. The time and distances were recorded every 30 s from transformational visual route of fish trajectory using Zebralab software.

**FIGURE 5:**
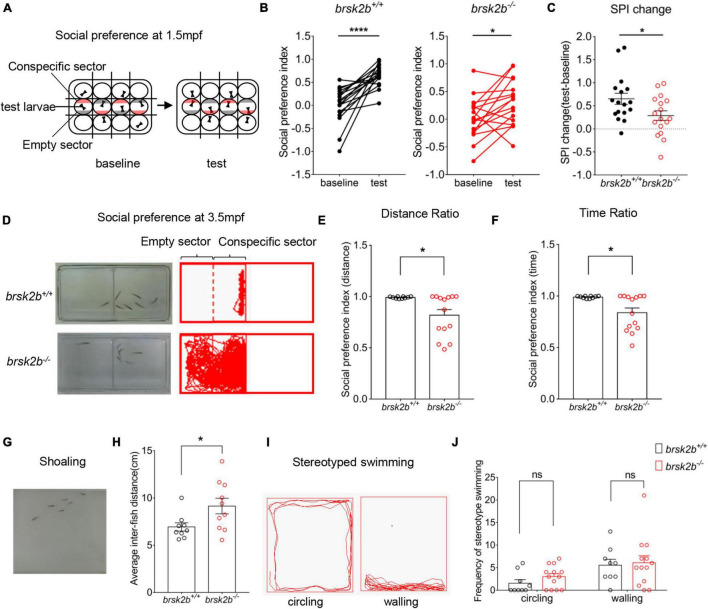
*brsk2b* mutant zebrafish displayed ASD-like behaviors. **(A,D)** The experimental paradigm of social preference test at 1.5 and 4 mpf. **(B,C)** The *brsk2b^–/–^* zebrafish displayed a significantly reduced preference for conspecifics compared to *brsk2b*^+/+^ zebrafish. And the change of social preference index (SPI) for *brsk2b^–/–^* zebrafish was significantly smaller than *brsk2b*^+/+^ fish (*brsk2b*^+/+^: *brsk2b^–/–^* = 17: 17, *p* = 0.029). Data for each genotype are presented as mean ± SEM and compared with Student’s *t*-tests. SPI changes are compared by a paired *t-*test. **(E,F)** The SPI of *brsk2b^–/–^* zebrafish at 4 mpf was significantly reduced compared to *brsk2b*^+/+^ fish both by distance **(E)** and time **(F)** ratio (*brsk2b*^+/+^: *brsk2b^–/–^* = 10: 14, *p* = 0.014 and 0.012, respectively). **(G)** The experimental paradigm of the shoaling test at 2.5 mpf. **(H)** The average inter-individual distance of *brsk2b^–/–^* zebrafish was smaller than that of *brsk2b*^+/+^ fish (*brsk2b*^+/+^: *brsk2b^–/–^* = 9: 10, *p* = 0.036). **(I)** The stereotyped swimming patterns in zebrafish are shown as “circling” and “walling.” **(J)** The *brsk2b^–/–^* zebrafish exhibited a trend of higher frequency of stereotyped behaviors though with no significant difference when compared to *brsk2b*^+/+^ fish (*brsk2b*^+/+^: *brsk2b^–/–^* = 9: 13, *p* = 0.206 and 0.980, respectively). Data for each genotype are presented as mean ± SEM and compared with Student’s *t*-tests. *p < 0.05, ****p < 0.0001.

For stereotyped behaviors, double-blind analyses to count the frequency of appearance of the “circling” and “walling” swimming pattern episodes within each minute were conducted separately (Figure 5I).

**FIGURE 6:**
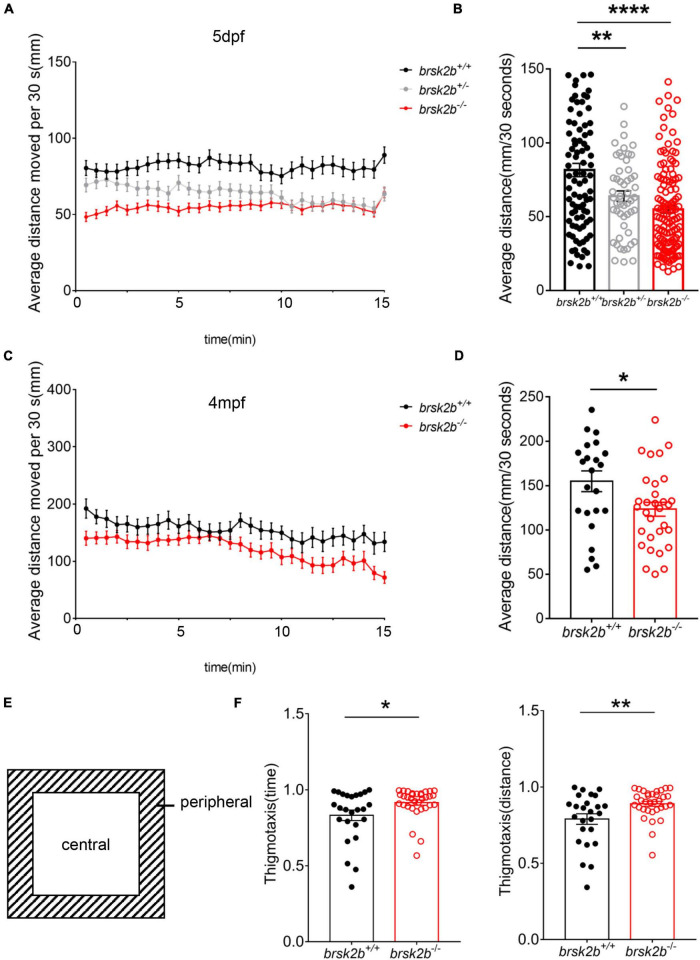
Locomotor analysis of *brsk2b*-deficient zebrafish showed impaired activity. **(A,B)** The activity of *brsk2b^–/–^* larvae was significantly reduced at 5 dpf compared to *brsk2b*^+/+^ larvae (*brsk2b*^+/+^: *brsk2b*^±^: *brsk2b^–/–^* = 89: 52: 128, *p* = 0.0001). **(C,D)** The activity of *brsk2b^–/–^* Adult zebrafish at 4 mpf also decreased than that of *brsk2b*^+/+^ fish (*brsk2b*^+/+^: *brsk2b^–/–^* = 24: 31, *p* = 0.024). **(E)** The tank was divided into two equal zones, the central zone and peripheral zone. The thigmotaxis was calculated by the time and distance ratio that fish spent in the peripheral zone. **(F)** The *brsk2b^–/–^* zebrafish tended to stay in the periphery that both the time and distance ratio of thigmotaxis were increased significantly (*brsk2b*^+/+^: *brsk2b^–/–^* = 25: 33, *p* = 0.023 and 0.007, respectively). Data are shown as mean ± SEM and compared by Student’s *t*-test for two genotypes and one-way ANOVA for three genotypes, *p < 0.05, **p < 0.01, ****p < 0.0001.

Thigmotaxis is a well-validated assay for anxiety in adult zebrafish (Kalueff et al., 2013). To analyze thigmotaxis, the open field tank was divided into the peripheral half and the central half (Figure 6E). Then the fish was placed to explore in the tank freely. Thigmotaxis was calculated as the ratio of time or distance that the fish spent in the peripheral zone, which followed the formula below:


$$\mathrm{Thigmotaxis}=\frac{\text{Time}/\mathrm{Ditance}\mathrm{spent}\mathrm{in}\mathrm{the}\mathrm{peripheral}\mathrm{zone}}{\text{Time}/\mathrm{Distance}\mathrm{spent}\mathrm{in}\mathrm{the}\mathrm{whole}\mathrm{zone}}$$

### Juvenile Zebrafish Social Behavior Tests

We conducted a social preference test with zebrafish at 1.5 mpf following a previously described experimental paradigm (Ruzzo et al., 2019). The experimental videos were also captured in a ViewPoint setup with automated video tracking system (Viewpoint Life Sciences). The fish were habituated in 12-well plates with removable opaque partitions (Figure 5A). The test fish were placed in the middle row of wells, with one fish per well. A single wild-type conspecific fish of similar age and size was placed in a well either above or below each middle well, with a corresponding opposite well empty. Each assay consisted of 5-min habituation before a 10-min baseline period and another 5-min habituation before a 10-min test period. In the baseline period, opaque partitions were inserted and divided every well to prevent fish from observing each other. Then during the test period, the opaque partitions between each row of wells were removed with the ones between each column of wells remaining, thus the test fish could only see conspecific well and empty well on both sides. When tracking the videos, the time zebrafish spent in both the quarter zone near the conspecific well and the quarter zone near the empty well was recorded (indicated as conspecific sector and empty sector, respectively, Figure 5A). For data analysis, social preference of test fish was measured by calculating the social preference index (SPI), which followed the calculation below:


$$\text{SPI}=\frac{\text{Time spent in conspecific sector}–\text{ Time spent in empty sector}}{\text{Time spent in both sectors}}$$

### Adult Zebrafish Social Preference Behavior Tests

To further assay social preference of *brsk2b* mutant zebrafish in adulthood, we performed another social experimental paradigm in mating tanks (dimensions 21 × 11 × 7.5 cm). The tank was separated into two equal zones by a transparent divider with a male test fish (wild-type or *brsk2b^–/–^*) at 3.5 mpf placed in one half and six male wild-type conspecifics of similar size and age placed in another, allowing them to see each other sufficiently (Figure 5D). Fish swam freely for 5-min acclimation before a 30-min video tracking. The time and distance were recorded every 30 s using Zebralab software. When tracking the videos, the half zone containing test fish was divided into two equal sectors, the conspecific sector close to 6 conspecifics and the empty sector that away from conspecifics (Figure 5D). The social preference was assessed by SPI and calculated as follows:


$$\text{SPI}=\frac{\text{Time}/\text{Distance in conspecific sector }-\text{ Time}/\text{Distance in empty sector}}{\text{ Time}/\text{Distance in both sectors}}$$

### Shoaling Tests

Adult fish form shoaling behavior that they aggregate with each other when swimming in a group of conspecifics. Six adult male zebrafish of the same genotype (wild-type or *brsk2b^–/–^*) at 2.5 mpf were placed in the novel tank apparatus for a 5-min habitation before the shoaling test (Figure 5G). Videos tracking fish swimming were recorded for 30 min. The shoaling behavior was quantified as the mean inter-individual distance that indicated the average of all distances between each zebrafish in a group.

### Statistical Analysis

Data was analyzed using GraphPad prism software. Normally distributed datasets were compared with the use of Student’s *t*-test and one-way ANOVA with Dunnett’s multiple comparisons tests. Values were presented as mean ± SEM. Data that were not normally distributed were analyzed as median ± 95% confidence interval using Kruskal–Wallis ANOVA with Dunn’s multiple comparisons tests. All statistical tests were 2-tailed, and statistical significance was defined as *p* < 0.05. *p < 0.05, ^**^p < 0.01, ^**^p < 0.001, ^****^p < 0.0001.

## Results

### Case Report and Literature Review

The proband was a 7-year-and-6-month-old boy, and first approached the Division of Child Health Care, Children’s Hospital of Fudan University at 19 months old due to failure to respond to his name being called. He showed distinct social defects manifesting as use of others’ hands as tools, barely responding when directly spoken to, failure to share enjoyment with others, little eye contact or use of body gestures, lack of interest in peers, and preferring solitary activities. Furthermore, he displayed restricted, repetitive patterns of behavior including indulgence in fiddling with round objects, especially wheels, and a narrow range of interest in strings, plastic bags, and neon lights. Moreover, he continued to present problems associated with hypotonia and gastrointestinal problems such as constipation, and had moderate feeding difficulties in the suckling period as reported by his parents.

**FIGURE 7:**
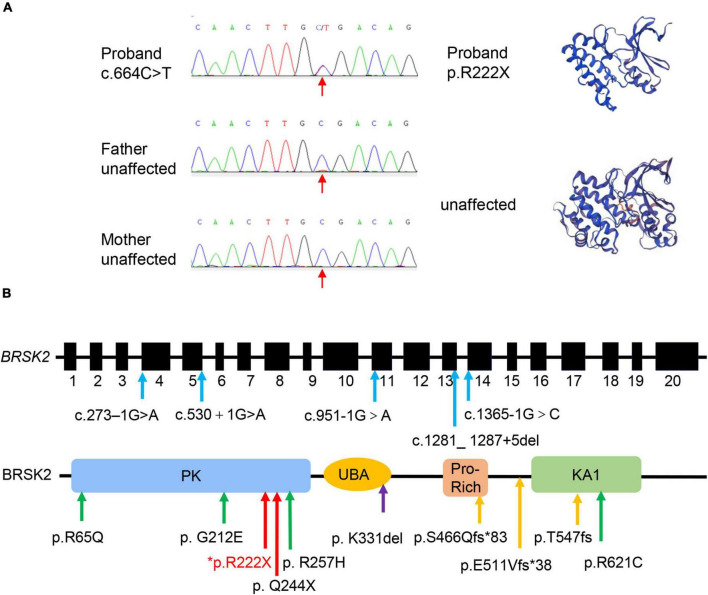
Genetic profile of *BRSK2*-deficient patients. **(A)** Sanger sequencing and computational modeling of BRSK2 in proband and unaffected parents. The red arrow indicates the mutation site. Proteins were modeled by SWISS-MODEL tool (https://swissmodel.expasy.org/). **(B)** Exon and domain structure and locations of observed variations in BRSK2. PK, protein kinase domain; UBA, ubiquitin-associated domain; Pro-Rich, proline-rich; KA1, kinase-associated domain. Blue arrow: splice site variation, green arrow: missense variation, yellow arrow: frameshift variation, red arrow: non-sense variation, purple arrow: microdeletion. * marks our proband.

His birth history was normal in that he was born at 40 weeks gestation and weighed 4,000 g. Due to pathological jaundice after birth, the proband received blue light treatment and stayed in the neonatal ward for 3 days. At the age of 8 months, he began to babble meaningless single syllables but still was not able to speak at first diagnosis. When he was 25 months old, he could only speak a few single words. He also presented motor developmental delay in that he could not walk independently until the age of 18 months. He was first evaluated at 1 year 7 months using the Griffiths Mental Development Scales (locomotor developmental quotient (DQ): 69; personal-social DQ: 46; hearing-speech DQ: 33; hand-eye coordination DQ: 59; performance DQ: 49) (Table 1), indicating global developmental delay. The results of the Autism Diagnostic Observation Schedule (ADOS) were 11 in social affect, 2 in restricted and repetitive behavior, and 13 in total score (ASD cut-off score: 11) (Table 1). Thus, this patient received a diagnosis of ASD based on the DSM-5 criteria and the standardized assessment of ADOS. A brain MRI and EEG showed nothing abnormal. His family history was unremarkable. Genetic evaluation of exome sequencing revealed a heterozygous non-sense mutation (p.R222X) in *BRSK2*, which leads to a premature translation termination codon and a 221-amino acid truncated protein (Figure 7A). This is a *de novo* variation confirmed by Sanger sequencing of the unaffected parents, and the variant was not found in the ExAC, 1000 Genomes, or ClinVar databases and has not been previously reported in the literature.

**TABLE 1:** Summary of *BRSK2* mutant patients’ early development and evaluation results.

Subjects	Our patient	^#^Patients published
Gender	Male	11/12, Male
Diagnosis of ASD	Yes	10/12, 1/12 Borderline
Speech delay	Yes	12/12
Motor delay	Yes	11/12
Intelligence disability	No	10/12
Age of walking (months)	18	12–20
Age of speaking (months)	25	18–24/non-verbal at evaluation
**Comorbidity**		
Feeding problem	Yes	2/12
Epilepsy	No	3/12
Sleep disorder	No	3/12
**Clinical evaluations**		
Age of first GMDS (months)	19.5	–
Locomotor DQ	69	
Personal-social DQ	46	
Hearing-speech DQ	33	
Hand-eye coordination DQ	59	
Performance DQ	49	
Age of WPPSI-IV (years, months)	4,6	–
VCI	88	
VSI	100	
FRI	89	
WMI	76	
PSI	86	
FSIQ	81	
Age of ADOS (months)	19.5	–
Social affect	11	
Restricted and repetitive behavior	2	
Total score	13 (ASD cut-off:11)	

*^#^Only 12 of 14 patients with detailed clinical data published in previous studies. GMDS, Griffiths Mental Development Scales. WPPSI-IV, Wechsler Preschool and Primary Scale of Intelligence-Fourth Edition. VCI, Verbal Comprehension Index; VSI, Visual Spatial Index; FRI, Fluid Reasoning Index; WMI, Working Memory Index; PSI, Processing Speed Index; FSIQ, Full Scale Intelligence Quotient. ADOS, Autism Diagnostic Observation Schedule, second edition.*

Through literature review, we identified 14 other cases with *BRSK2* variants in DD/ID or ASD cohorts (1 microdeletion, 2 non-sense variants, 3 frameshift variants, 4 missense variants, and 5 variants affecting splice sites; Figure 7B). Twelve of the 14 cases reported detailed clinical data (Table 1 and Supplementary Table 2). We compared the phenotypic features of our patient and these 12 cases. Twelve of the 13 cases (92.3%) were male. Their ages ranged from 3 years 8 months to 19 years (mean: 8 years 5 months; Supplementary Table 2). ASD was the most common diagnosis observed in these subjects, with 12 diagnosed (92.3%) and one considered borderline. All patients presented with speech delay (100%, 13/13), 11 presented with motor delay (84.6%, 11/13), and 10 displayed mildly to severely impaired intelligence (76.9%, 10/13). In addition, some were reported to have comorbidities, including sleep disorders (23.1%, 3/13), feeding problems (15.4%, 2/13), and ADHD (23.1%, 3/13) (Table 1). Details are presented in Supplementary Table 2.

### BR Serine/Threonine Kinase 2 Is a Conserved Gene in the Vertebrate Lineage

The zebrafish *brsk2a* gene is located on chromosome 25 and has 20 exons, and the *brsk2b* gene is located on chromosome 7 and has 21 exons (Figure 1A). To analyze the evolutionary conservation of BRSK2 between human and zebrafish, we performed protein sequence alignments of these two species in detail. The results showed that both the brsk2a and brsk2b proteins exhibited a high level of amino acid identity with human BRSK2 (85.47 and 89.59%, respectively; Table 2). In particular, the protein kinase domains and UBA domains of both brsk2a and brsk2b were highly concordant with those of human BRSK2 (96.41 and 97.13% for the PK domain, and 86.05 and 83.72% for the UBA domain, respectively; Table 2). Therefore, zebrafish is a good model for studying the function of the human BRSK2 protein and its association with ASD.

**TABLE 2:** Conservation analysis of domains and proteins of BRSK2 between human and zebrafish.

Protein alignment	Conserved level of protein	Conserved level of PK domain	Conserved level of UBA domain
BRSK2 VS. brsk2a	85.47%	96.41%	86.05%
BRSK2 VS. brsk2b	89.59%	97.13%	83.72%
BRSK2A VS. brsk2b	86.78%	97.95%	96.30%

*The analysis was performed by DNAMAN software (version 9.0.1.116).*

To further identify orthologs between zebrafish *brsk2* and other species, we built a phylogenetic tree to conduct phylogenetic analysis (Figure 1B). The numbers on the phylogenetic tree represent evolutionary relationships, with larger numbers indicating larger genetic differences. In vertebrate animals, the genetic distance was not greater than 0.1; however, in invertebrates (such as *Caenorhabditis elegans*), the distance was as high as 0.8 (Figure 1B). Therefore, this phylogenetic analysis indicated that *BRSK2* has high evolutionary conservation among vertebrates.

**FIGURE 8:**
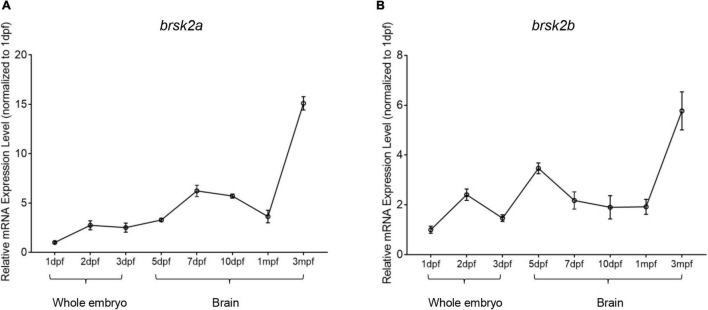
Temporal mRNA expression profiling of zebrafish *brsk2*. The mRNA expression levels of *brsk2a* **(A)**, *brsk2b* **(B)** at 8 stages (Data are shown as mean ± SEM, *n* = 3 for each genotype). mRNA was extracted from whole embryos of 1 dpf, 2 dpf, 3 dpf, and brain tissues of 5 dpf, 7 dpf, 10 dpf, 1 mpf, and 3 mpf.

Previous research showed that *Brsk2* is mainly expressed in the cerebrum in rodents (Kishi et al., 2005). To determine the temporal and developmental expression patterns of *brsk2a* and *brsk2b* in zebrafish, RT-qPCR was performed to verify *brsk2* expression levels of wild-type zebrafish at eight developmental stages. The results showed that the expression levels of *brsk2a* and *brsk2b* generally increased with development. Expression of *brsk2a* began at a very early stage (1 dpf) and remained at a relatively low level during the larval period, following a slow increase before reaching its highest level in adulthood (3 mpf) (Figure 8A). The expression level of *brsk2b* was relatively high at 2 dpf, subsequently decreasing at 3 dpf and increasing at 5 dpf, then displayed another increasing peak between 1 mpf and adulthood (Figure 8B). In summary, these data indicate that both *brsk2a* and *brsk2b* are expressed during development and continue to be enriched in the mature central nervous system.

### Generation of *brsk2b* Mutant Zebrafish

To determine *BRSK2* function *in vivo*, we generated a loss-of-function variant of *brsk2b* in zebrafish using CRISPR/Cas9 mutagenesis technology (Auer et al., 2014). The *brsk2b^–/–^* mutant harbors a 2-basepair (GG) insertion resulting in a frameshift mutation and a 143-amino acid truncated protein (Figures 2A,B). RT-qPCR showed that mRNA expression levels of *brsk2b* were dramatically decreased in mutant zebrafish (Figure 2C), while *brsk2b* deficiency did not affect the mRNA expression level of *brsk2a* (Figure 2D). Therefore, these results demonstrate that the *brsk2b* mutant zebrafish line was successfully generated.

### The *brsk2b* Mutant Larvae Display Developmental Delay at Early Stages

As previous studies demonstrated that *BRSK2* is essential to early neurogenesis in rodents (Kishi et al., 2005; Nakanishi et al., 2019), we analyzed the morphological characteristics of *brsk2b* mutant zebrafish larvae in early developmental stages (1–3 dpf). Compared with *brsk2b^+/+^* and *brsk2b*^±^ larvae, a significantly larger proportion of *brsk2b^–/–^* larvae died at 24 hpf (65.9%) and manifested pericardium edema, severe developmental delay, and depigmentation at 48 hpf (26.1%) and 72 hpf (9.1%) (Table 3). The *brsk2b^–/–^* and *brsk2b*^±^ larvae displayed much shorter body lengths than *brsk2b^+/+^* larvae at 2 dpf (*brsk2b^+/+^*: 3.869 ± 0.06 mm; *brsk2b*^±^: 3.728 ± 0.09 mm; *brsk2b^–/–^*: 3.709 ± 0.13 mm, *p* < 0.0001), 3 dpf (*brsk2b^+/+^*: 4.567 ± 0.09 mm; *brsk2b*^±^: 4.376 ± 0.12 mm; *brsk2b^–/–^*: 4.387 ± 0.09 mm, *p* < 0.0001), and 5 dpf (*brsk2b^+/+^*: 4.899 ± 0.09 mm; *brsk2b*^±^: 4.820 ± 0.13 mm; *brsk2b^–/–^*: 4.808 ± 0.08 mm, *p* = 0.0043 and 0.0145, respectively), which indicated a pattern of growth retardation (Figures 3C–E). We also measured the value of OT, which is correlated with width of the head, and found that *brsk2b*^±^ and *brsk2b^–/–^* larvae exhibited smaller OT at 5 dpf compared to *brsk2b^+/+^* larvae (*brsk2b^+/+^*: 0.650 ± 0.01 mm; *brsk2b*^±^: 0.636 ± 0.01 mm; *brsk2b^–/–^*: 0.632 ± 0.02 mm, *p* = 0.0028 and 0.00001, respectively; Figure 3F). However, there was no significant difference of the ratio of OT/body length among the three lines (*brsk2b^+/+^*: 0.133 ± 0.002; *brsk2b*^±^: 0.132 ± 0.003; *brsk2b^–/–^*: 0.131 ± 0.004, *p* = 0.648 and 0.287, respectively; Figure 3G).

**TABLE 3:** Mortality and morphological analysis in early development.

	*brsk2b^+/+^*	*brsk2b* ^+/−^	*brsk2b^–/–^*
Mortality at 24 hpf	2/92 (2.2%)	8/59 (13.6%)	54/82 (65.9%)
^#^Abnormal development at 48 hpf	0/30 (0%)	5/27 (18.5%)	6/23 (26.1%)
^#^Abnormal development at 72 hpf	2/40 (5%)	10/47 (21.2%)	2/22 (9.1%)

***^#^**Abnormal development includes pericardium edema, severe developmental delay and depigmentation.*

### *brsk2b* Mutant Zebrafish Exhibit Impaired Locomotor Activity in Both the Larval Phase and Adulthood

As most *BRSK2* mutant patients show motor delays, locomotor activity was measured in both the larval and adult zebrafish. During the 15-min observation, the larvae at 5 dpf displayed a significant decrease in the average swimming velocity of *brsk2b^–/–^* zebrafish (54.86 ± 29.48 mm/30 s) and *brsk2b*^±^ zebrafish (63.80 ± 25.81 mm/30 s) compared to *brsk2b^+/+^* zebrafish (81.52 ± 42.07 mm/30 s, *p* = 0.0001 and 0.0053, respectively; Figures 6A,B). Similarly, a significantly reduced swimming velocity was observed in *brsk2b^–/–^* fish at 4 mpf compared with *brsk2b^+/+^* fish (*brsk2b^–/–^* vs. *brsk2b^+/+^.*: 123.4 ± 43.64 mm/30 s vs. 155.0 ± 57.29 mm/30 s, *p* = 0.0241; Figures 6C,D). Moreover, *brsk2b^–/–^* zebrafish showed steadily lower locomotor activity throughout the examination window.

In the analysis of the light/dark test, the average swimming distance of *brsk2b^–/–^* larvae during each 5-min light or dark period was dramatically lower (D1: 90.91 ± 25.056 mm/30 s, *p* < 0.0001; L1: 18.144 ± 15.271 mm/30 s, *p* < 0.0001; D2: 64.259 ± 22.252 mm/30 s, *p* < 0.0001; L2: 15.318 ± 13.194 mm/30 s, *p* < 0.0001; D3: 57.902 ± 21.509 mm/30 s, *p* < 0.0001, L3: 13.126 ± 12.103 mm/s, *p* < 0.0001; Figures 4A,B) than *brsk2b^+/+^* and *brsk2b*^±^ larvae, which provides additional evidence of impaired locomotor phenotypes in *brsk2b^–/–^* fish.

We also examined the responses evoked by light-dark switching. In general, light-to-dark transitions elicit sudden increases in swimming velocity, while dark-to-light transitions result in sudden decreased velocity, which is called the visual motor response (Burton et al., 2017). As reported, a sudden increase evoked by the illumination conversion was used to represent the anxiety level of the zebrafish (Krylov et al., 2021). During three light-to-dark transitions, the *brsk2b*^–/–^ zebrafish at 5 dpf exhibited a trend of enhanced movement with a higher velocity change compared to the other lines [*brsk2b^+/+^*: 0.62 (95% CI: 0.816–1.451); *brsk2b*^±^: 0.82 (95% CI: 0.840–1.605); *brsk2b^–/–^*: 1.01 (95% CI: 1.13–1.67); Figures 4C,D] during the 30 s before and after the light was turned off. Similarly, in three dark-to-light transitions, the velocity change of *brsk2b*^–/–^ larvae was more dramatic than that of the other lines [*brsk2b^+/+^*: 0.47 (95% CI: 0.401–0.492); *brsk2b*^±^: 0.60 (95% CI: 0.560–0.644); *brsk2b^–/–^*: 0.68 (95% CI: 0.616–0.679); Figures 4E,F]. Our analysis found that *brsk2b^–/–^* larvae exhibited a larger velocity change when the lights were turned off and on, which might indicate that *brsk2b^–/–^* larvae present more stress when illumination changes. In addition, thigmotaxis was also measured in adult zebrafish to analyze anxiety-like behaviors. The results showed that *brsk2b^–/–^* zebrafish at 4 mpf spent more swimming time and distance in the peripheral zone (0.92 ± 0.02 for time ratio and 0.89 ± 0.02 for distance ratio) compared to *brsk2b^+/+^* zebrafish (0.83 ± 0.03 for time ratio and 0.79 ± 0.03 for distance ratio, Figure 6F). In summary, our work demonstrates that *brsk2b^–/–^* zebrafish had higher anxiety.

### *brsk2b* Mutant Zebrafish Display Autism Spectrum Disorder-Like Behaviors

To assay whether *brsk2b* mutant zebrafish displayed ASD-like characteristics, we performed multiple tests of ASD-like behaviors from juvenile to adult zebrafish. We first performed a modified social preference test on 1.5 mpf juvenile zebrafish (Figure 5A). The results showed that *brsk2b^+/+^* zebrafish generally spent more time in the conspecific sector rather than the empty sector, implicating a strong group tendency. In contrast, *brsk2b^–/–^* fish exhibited a reduced duration and frequency of social contact with peer wild-type fish, and their SPI change between the baseline period and the test period was significantly decreased compared to *brsk2b^+/+^* zebrafish, indicating that juvenile *brsk2b^–/–^* zebrafish showed a significant social deficit (Figures 5B,C). Similarly, in the adult (3.5 mpf) social preference assay, *brsk2b^–/–^* fish spent their time evenly throughout the region and displayed a reduced duration and frequency of social contact with the peer group compared to *brsk2b^+/+^* fish, indicating a significantly decreased social preference (Figures 5E,F). The shoaling test is another important social behavioral test. In the shoaling assay, *brsk2b^+/+^* zebrafish tended to aggregate together and showed much smaller inter-fish distances, while the *brsk2b^–/–^* zebrafish swam more separately and displayed larger and looser schools, as well as much larger inter-fish distances (Figure 5H). The analysis of trajectories of activity and patterns of swimming showed that *brsk2b^–/–^* zebrafish exhibited a trend of a higher frequency of stereotyped behaviors, although they were not significantly different to *brsk2b^+/+^* fish (Figure 5J).

### *brsk2b* Disruption Affects Normal Neurodevelopment in Mutant Zebrafish

To identify potential mechanisms underlying ASD-like behaviors, we first assessed the impact of neurodevelopment *in vivo*, as a previous study observed that *Brsk1/Brsk2* double mutant mice displayed aberrant cortex development (Kishi et al., 2005). We crossed *brsk2b^–/–^* zebrafish with a Tg (*HuC*: RFP) transgenic line in which RFP protein expression is specifically controlled by the neural *HuC* promoter, allowing the visualization of neurodevelopment at embryonic stages. As shown in Supplementary Figure 1, the RFP signal intensities of *brsk2b^–/–^* larvae at 1, 2, and 3 dpf were dramatically decreased compared to *brsk2b^+/+^* larvae, indicating a deleterious effect on neurodevelopment.

**FIGURE 9:**
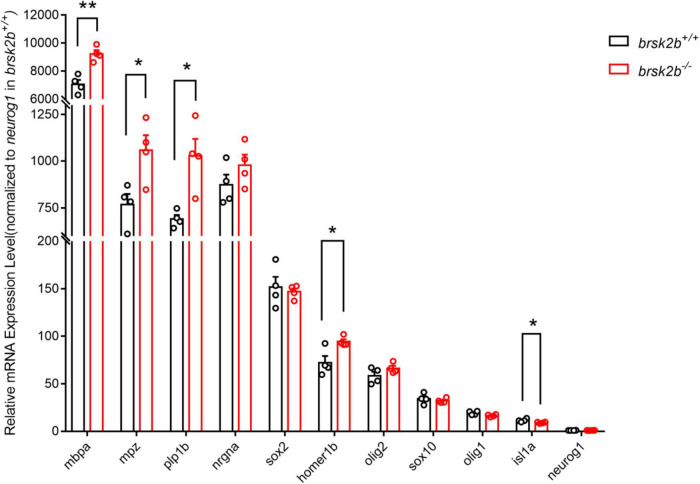
RT-qPCR showed altered expression levels of neurogenesis-related markers in *brsk2b^–/–^* zebrafish. Human homologous protein: HOMER1, Homer Scaffold Protein 1, a postsynaptic density scaffolding protein; NRGN, Neurogranin, a postsynaptic protein kinase substrate; ISL1, a DNA-binding transcriptional activator; SOX2, a transcription factor regulating embryonic development and determining cell fate; NEUROG1, a transcriptional regulator involved in the initiation of neuronal differentiation; OLIG1, Oligodendrocyte Transcription Factor 1, promotes formation and maturation of oligodendrocytes; OLIG2, Oligodendrocyte Transcription Factor 2, required for oligodendrocyte and motor neuron specification in the spinal cord; SOX10, a transcription factor that plays a central role in developing and mature glia; MBP, a major constituent of the myelin sheath of oligodendrocytes and Schwann cells in the nervous system; MPZ, a major structural protein of the peripheral myelin sheath; PLP, a major myelin protein in central nervous system. *n* = 4 for each genotype, data for each gene are shown as mean ± SEM and compared with multiple *t*-tests. *p < 0.05, **p < 0.01.

In addition, we examined the mRNA expression levels of a group of markers involved in neurogenesis, including a postsynaptic density scaffolding protein (homer1b), general neurogenesis proteins (nrgna, neurog1, and sox2), a motor neuron-specific protein (isl1a), and oligodendrocyte-related proteins (olig1, olig2, sox10, mbpa, mpz, and plp1b). When normalized to the expression level of *neurog1* in wild-type zebrafish, the results showed that the mRNA expression levels of *homer1b* (*brsk2b^+/+^*: *brsk2b^–/–^* = 72.17 ± 7.082: 94.41 ± 2.547, *p* = 0.025), *mbpa* (*brsk2b^+/+^*: *brsk2b^–/–^* = 7,064 ± 317.6: 9,229 ± 265.7, *p* = 0.002), *mpz* (*brsk2b^+/+^*: *brsk2b^–/–^* = 768.8 ± 55.54: 1,058 ± 80.27, *p* = 0.025), and *plp1b* (*brsk2b^+/+^*: *brsk2b^–/–^* = 691.4 ± 22: 1,028 ± 90.76, *p* = 0.011) in *brsk2b^–/–^* zebrafish were significantly increased compared to *brsk2b^+/+^* zebrafish, while the expression level of *isl1a* was clearly decreased (*brsk2b^+/+^*: *brsk2b^–/–^* = 11.5 ± 0.834: 9.08 ± 0.345, *p* = 0.037). Otherwise, no significant differences were observed in the levels of *neurog1* (*brsk2b^+/+^*: *brsk2b^–/–^* = 1 ± 0.005: 0.98 ± 0.068, *p* = 0.794), *nrgna* (*brsk2b^+/+^*: *brsk2b^–/–^* = 873.1 ± 54.31: 978.5 ± 56.39, *p* = 0.2271), *sox2* (*brsk2b^+/+^*: *brsk2b^–/–^* = 151.7 ± 10.83: 146.8 ± 3.559, *p* = 0.680), *olig1* (*brsk2b^+/+^*: *brsk2b^–/–^* = 19.31 ± 1.003: 16.54 ± 0.634, *p* = 0.059), *olig2* (*brsk2b^+/+^*: *brsk2b^–/–^* = 58.48 ± 4.209: 66.26 ± 2.634, *p* = 0.168), and *sox10* (*brsk2b^+/+^*: *brsk2b^–/–^* = 34.23 ± 2.73: 32.15 ± 1.223, *p* = 0.514) (Figure 9). These data illustrate that the neurodevelopment of *brsk2b^–/–^* zebrafish might be affected through disturbed synaptogenesis, oligodendrocyte maturation, and myelin sheath formation.

## Discussion

Here, we report a novel *BRSK2* non-sense variant in a patient with ASD. The patient displayed distinct social defects and a narrow range of interests with mild developmental delay in language and motor skills. To determine the correlation between genotype and phenotype, we established *brsk2b* mutant zebrafish with CRISPR/Cas9 technology. Morphological assessment revealed that *brsk2b* mutant larvae exhibited higher embryonic lethality and rate of pericardium edema, severe developmental delay and depigmentation as well as growth retardation. The *brsk2b* mutant zebrafish displayed ASD-like behaviors and showed locomotor dysfunction in both the juvenile and adult stages. In addition, we found that the mRNA expression level of *homer1b, mbpa, mpz*, and *plp1b* were increased, while *isl1a* was decreased in *brsk2b* mutant zebrafish, indicating disrupted postsynaptic structure and oligodendrocyte function. In summary, we conclude that *brsk2b* dysfunction in zebrafish induced early developmental delay and behavioral abnormalities similar to those seen in autism.

The patient in this study was diagnosed with ASD for social defects and manifested speech and motor delays, which were concordant with the symptoms of *BRSK2* patients reported in previously published literature (Feliciano et al., 2019; Hiatt et al., 2019). Exome sequencing data revealed that the patient had a *de novo* non-sense variant in *BRSK2* (p.R222X), which was predicted to be deleterious. Previous publications reported that 14 described non-sense, splice, frameshift, and predicted-deleterious missense *BRSK2* variations were likely responsible for the phenotypes of these DD/ID patients with or without ASD (Miller et al., 2010; Kaminsky et al., 2011; Feliciano et al., 2019; Hiatt et al., 2019). Therefore, these cases indicate that *BRSK2* is relatively intolerant to protein-altering variation and plays a role in neurological diseases including ASD.

BRSK2 is highly evolutionarily conserved in vertebrates, and both the brsk2a and brsk2b protein in zebrafish exhibit a high level of amino acid identity with human BRSK2. A recent study demonstrated that *Brsk2*-deficient mice showed neonatal lethality and a disorganized cortex after being backcrossed with C57BL/6 mice for at least six generations, which was not observed in *Brsk1*-deficient mice, indicating that *Brsk2* plays a more essential role in neurodevelopmental and cortical process in rodents (Nakanishi et al., 2019). In our study, *brsk2b*-deficient zebrafish exhibited a higher embryonic mortality rate and dysplasia including pericardium edema, severe developmental delay, and depigmentation when compared to wild-type zebrafish. Though non-negligible embryonic lethality was observed in some individuals, the *brsk2b*-deficient zebrafish were able to survive to adulthood and were fertile. The duplication of the *brsk2* gene in zebrafish may account for this difference between *Brsk2*-deficient mice and zebrafish in that *brsk2a* may perform similar functions as *brsk2b*. Notwithstanding, our RT-qPCR experiment did not reveal any compensational increase of *brsk2a* mRNA, but we speculate that the function of *brsk2a* was retained after *brsk2b* was defective, which may compensate for some of the phenotypes. Furthermore, *brsk2b*-deficient larvae had shorter body lengths than wild-type larvae at 2, 3, and 5 dpf. This observation was also reminiscent of the growth retardation observed in a *Brsk2* knockout mouse model (Nie et al., 2013). Although the value of OT, representing the brain size of *brsk2b* mutant larvae, was smaller than those of wild-type larvae at 5 dpf, the ratio of OT to body length did not show any difference. Particularly, our observation of weaker RFP signals in the neural system of Tg (*huC*: RFP); *brsk2b* mutant larvae demonstrated that *brsk2b* dysfunction disturbed neurodevelopment. Previously cultured hippocampal and cortical neurons from *Brsk1/Brsk2* double knockout mice *in vitro* exhibited disrupted polarization and a large proportion of indeterminate neurites, which were positive for both axonal and dendritic markers (Muller et al., 2010; Dhumale et al., 2018). And *Brsk1/Brsk2* double mutant mice showed reduced cortical axon tracts and displayed a much thinner cortex in which the apoptosis of neurons and a reduction of progenitors were detected (Kishi et al., 2005; Dhumale et al., 2018). Together, our observations, which echo findings in rodent animals, provide additional evidence that brsk2b dysfunction affects embryonic neurodevelopment.

Locomotion is the most basic and complex motor behavior in vertebrates which is controlled by neural activity (Grillner and El Manira, 2020). Disrupted neural structures and dysregulated brain development may affect the locomotor behavior of zebrafish larvae (Liu et al., 2022). In the activity test, *brsk2b^–/–^* larvae demonstrated hypoactivity, with their average swimming distance per 30 s being much shorter than that of *brsk2b*^±^ and *brsk2b^+/+^* larvae. This aberrant motor pattern remained during the adult stage, with *brsk2b^–/–^* zebrafish at 4 months old also displaying lower average movement compared to their wild-type counterparts in open field tests. These discoveries are analogous to patients with *BRSK2* variants in that 84.6% of reported cases (11/13) experienced motor developmental delays at an early age and exhibited motor function impairments thereafter (Feliciano et al., 2019; Hiatt et al., 2019). Although *Brsk2* null mice studies did not show detailed data on behavioral tests (Kishi et al., 2005; Nakanishi et al., 2019), these mice exhibited little spontaneous movement (Kishi et al., 2005), and the neuromuscular junction of conditional *Brsk1/Brsk2* null mice had disorganized neurofilaments and required BRSK kinases presynaptically for synaptic maturation (Lilley et al., 2014). Thus, our work suggests that *brsk2* deficiency impairs the motor function of *brsk2b* mutant zebrafish. In addition, further detection of the mRNA level revealed that the expression of *isl1a*, a motor neuron specific gene, was reduced, which might underlie the motor defects in *brsk2b* mutant fish.

It is known that anxiety is one of the comorbid symptoms in ASD (Meshalkina et al., 2018) and one of the reported patients with *BRSK2* variation (p. Gln244X) was diagnosed with anxiety disorder, so we evaluated whether this phenotype was displayed in *brsk2b* mutant zebrafish in behavioral tests. In nature, the response to the conversion in illumination reflects an aversion of darkness, which is a survival instinct to escape predators casting shadows on zebrafish larvae (Mahabir et al., 2013). Thus, sudden increased activity evoked by light changes reflects a state of stress, and this visual motor response is considered an index of anxiety (Ellis et al., 2012; Schnorr et al., 2012; Peng et al., 2016; Basnet et al., 2019). While all the three genotypes of *brsk2b* larvae at 5 dpf showed sudden increased activity provoked by dark, *brsk2b^–/–^* larvae exhibited a more remarkable transient velocity change between the 30 s before and after the light-to-dark and dark-to-light transition. Since the average activity of *brsk2b^–/–^* larvae in both the light and dark phases was lower than that of *brsk2b*^±^ and *brsk2b^+/+^* larvae, we speculate that *brsk2b^–/–^* larvae displayed anxiety-like behavior. On the other hand, thigmotaxis is a well-validated index of anxiety that is evolutionarily conserved and is displayed by a wide range of species (Treit and Fundytus, 1988). Thigmotaxis (also called “wall-hugging” or “wall-following” behavior) is the propensity to avoid the center of an arena and stay or move in close proximity to the boundaries of a novel environment (Sharma et al., 2009; Schnorr et al., 2012). In our study, the adult *brsk2b^–/–^* zebrafish reduced the exploration of the central area and tended to spend more time in the periphery, which was presented as enhanced thigmotaxis, suggesting adult *brsk2b^–/–^* zebrafish also exhibited a more anxious state.

Although phenotyping and genotyping assessments of patients implicate damaging variants in *BRSK2* as contributing to autism, sufficient evidence is lacking to further verify the correlation between *BRSK2* and ASD and its underlying molecular mechanism based on animal models. To fill in this gap, we conducted several behavioral tests to evaluate whether *brsk2b* mutant zebrafish exhibit ASD-like features. The results showed that *brsk2b^–/–^* zebrafish exhibited significant aberrant behavior compared to *brsk2b^+/+^*zebrafish. In both juveniles and adults, in the social preference test, *brsk2b^–/–^* zebrafish exhibited social impairment as some of their preference to conspecifics reduced significantly compared to *brsk2b^+/+^* fish. In addition, *brsk2b* mutant zebrafish swam loosely in a group, while *brsk2b^+/+^* zebrafish aggregated with each other, indicating that *brsk2b* dysfunction impacted the sociality of *brsk2b* mutant fish. Moreover, *brsk2b* mutant zebrafish tended to swim in stereotyped behavioral patterns such as “circling” and “walling” in the open field test, although there was no statistically significant difference compared to wild-type zebrafish. In conclusion, *brsk2b* mutant zebrafish displayed ASD-like behaviors.

To further understand the underlying molecular mechanisms of alterations in the morphology and behavior of *brsk2b*-deficient zebrafish, we detected the mRNA expression level of genes related to neurogenesis. The results showed that the expression levels of *homer1b, mbpa, mpz*, and *plp1b* in *brsk2b^–/–^* zebrafish were significantly increased while the *isl1a* distinctly decreased. Human HOMER1 is a postsynaptic density scaffolding protein that is highly expressed in the hippocampus, corpus striatum, and cortex, which regulates the morphogenesis of synapses and dendritic spines (Yoon et al., 2021). The up-regulated expression of *homer1b* in *brsk2b^–/–^* zebrafish indicates that these processes relevant to synaptogenesis may be disrupted. ISL1 is restricted in the motor neurons and is required for various phases of motor neuron development (Kim et al., 2015). *Isl1* mutant mice manifest motor function disabilities and have impaired axonal trajectories and axon specifications for motor neurons (Liang et al., 2011). As BRSK kinases were also identified as regulating synapse maturation at neuromuscular junctions (Lilley et al., 2014), we speculate that brsk2b might correspond to isl1a in motor neuron function; thus, *brsk2b* deficiency led to the disabled locomotor function of *brsk2b* mutant zebrafish. Further research on the roles of *isl1* in *brsk2*-affected motor function would be helpful to elucidate the mechanism of motor disorder in *BRSK2* patients. The human MBP protein is a critical component of myelin (Herbert et al., 2017), and MPZ is expressed exclusively in Schwann cells. Mutations in *MPZ* were associated with the peripheral neuropathy Charcot-Marie-Tooth disease type 1 (Sanmaneechai et al., 2015). The *PLP1* gene plays a critical role in oligodendrocyte development and myelin sheath formation by promoting sheath compaction (Nobuta et al., 2019). Actually, large previous studies have linked oligodendrocyte dysfunction with ASD (Galvez-Contreras et al., 2020). Magnetic resonance imaging detected myelination defects and an abnormal morphology of white matter in ASD patients (Deoni Zinkstok et al., Deoni Zinkstok et al.; Aoki et al., 2017). Similarly, animal models of ASD displayed myelination dysfunction and disturbed regulation of oligodendrocyte-specific genes (Kawamura et al., 2020; Phan et al., 2020). Altogether, the expression changes of these genes indicate that *brsk2* deficiency may disturb the processes of oligodendrocytes maturation and myelin sheath formation and lead to morphological and behavioral abnormalities that are relevant to neurological disorders and ASD.

Since the *BRSK2* gene is duplicated as *brsk2a* and *brsk2b* in zebrafish and these genes have highly conserved amino acid sequences, we speculate that *brsk2a* plays a parallel role to *brsk2b*, thus it may compensate for the function of *brsk2b* when it is mutated. Therefore, a *brsk2a* and *brsk2b* double mutant zebrafish model is necessary to provide further support to the correlation between *BRSK2* and ASD occurrence. In particular, our research preliminarily found that *brsk2b* deficiency affected oligodendrocyte and synaptic structure-related genes. Therefore, in future studies, the function of oligodendrocyte lineage cells and molecular pathways relevant to synaptogenesis involved in *BRSK2* and autism should be examined based on a *brsk2a/brsk2b* double mutant zebrafish model, hoping to find new approaches and potential drugs for the treatment of ASD.

In this study, we identified a proband with a *de novo* non-sense variant in *BRSK2* (p.R222X) exhibiting typical ASD behaviors. For the first time, this work provided a new genetic model of *brsk2b* mutant zebrafish, which displayed impaired social preferences and disturbed shoaling behaviors as well as distinct locomotor defects. The increased expression level of *homer1b, mbpa, mpz*, and *plp1b* suggests that these ASD-like phenotypes may be involved in postsynaptic structure and oligodendrocyte and myelin sheath function, which remain to be further studied in future work.

## Data Availability Statement

The original contributions presented in the study are included in the article/Supplementary Material, further inquiries can be directed to the corresponding authors.

## Ethics Statement

The studies involving human participants were reviewed and approved by the Research Ethics Board of Children’s Hospital of Fudan University. Written informed consent to participate in this study was provided by the participants’ legal guardian/next of kin. The animal study was reviewed and approved by the Research Ethics Board of Children’s Hospital of Fudan University.

## Author Contributions

CL and XX conceived and supervised the study. XX, CL, and JD designed the experiments. JD performed the experiments and analyzed the data. JD, YW, and MH provided homozygous identification. QL and JL provided technical assistance in behavioral tests. JD and CL wrote the manuscript. All authors contributed to the article and approved the submitted version.

## Conflict of Interest

The authors declare that the research was conducted in the absence of any commercial or financial relationships that could be construed as a potential conflict of interest.

## Publisher’s Note

All claims expressed in this article are solely those of the authors and do not necessarily represent those of their affiliated organizations, or those of the publisher, the editors and the reviewers. Any product that may be evaluated in this article, or claim that may be made by its manufacturer, is not guaranteed or endorsed by the publisher.

## Footnotes

1
http://www.ncbi.nlm.nih.gov/gene/

2
https://swissmodel.expasy.org/
