# Supplementary material for: Deleterious Variation in BR Serine/Threonine Kinase 2 Classified a Subtype of Autism
Source: Front Mol Neurosci. 2022 Jun 10;15:904935. doi: 10.3389/fnmol.2022.904935 (PMC9231588; doi:10.3389/fnmol.2022.904935)
Supplement: Supplementary file 1 [file Data_Sheet_1.docx]

**
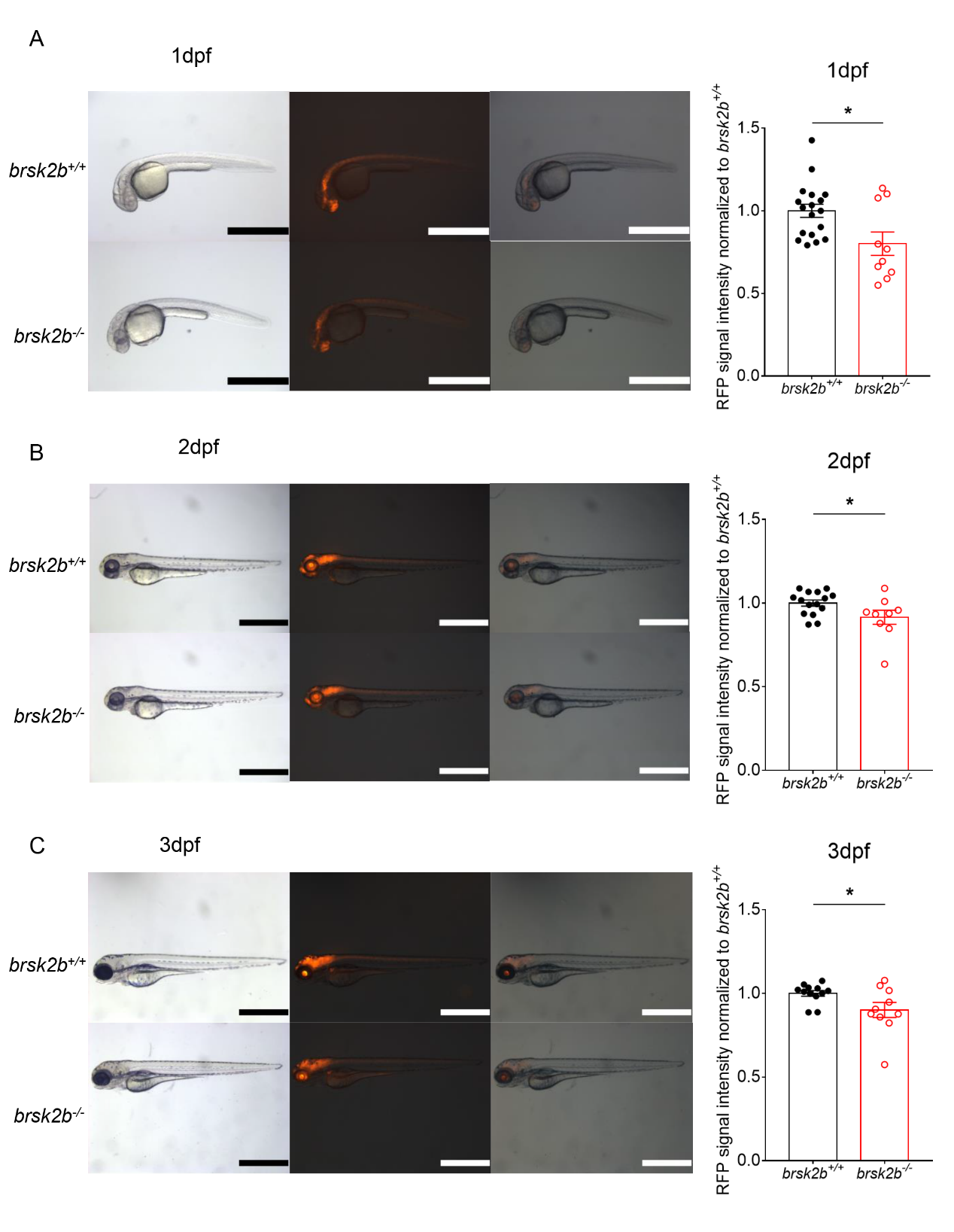
**

**Supplementary Figure 1. *brsk2b* disruption destroyed normal neurodevelopment.** Tg (*HuC*: RFP) zebrafish and Tg (*HuC*: RFP); *brsk2b^-/-^* zebrafish larvae were pictured laterally on a Leica 205C fluorescence microscope, scale bars 1mm. The RFP signal in *brsk2b^-/-^* larvae was reduced significantly at 1dpf **(A)**,2dpf **(B)**,3dpf **(C)**. (*brsk2b^+/+^*: *brsk2b^-/-^* =18: 10; 15: 9; 12: 9, respectively.) Data are presented as mean ± SEM and compared with student’s *t*-tests, * p < 0.05.
