# Supplementary material for: Deleterious Variation in BR Serine/Threonine Kinase 2 Classified a Subtype of Autism
Source: Front Mol Neurosci. 2022 Jun 10;15:904935. doi: 10.3389/fnmol.2022.904935 (PMC9231588; doi:10.3389/fnmol.2022.904935)
Supplement: Supplementary file 2 [file Table_1.docx]

**Supplementary Table 1**

gRNA target site, primers for genotyping and RT-qPCR

| Item | Oligo name | Sequence (5'-3') | Amplicon length(bp) | gene ID |
| --- | --- | --- | --- | --- |
| *brsk2b* gRNA target site | *brsk2b*-gRNA-E4 | GGGCAGGTTAACACCCAAAG |  | NC_007118.7 |
| PCR |  |  |  |  |
| *brsk2b* genotyping | *brsk2b*-gRNA-I3-PCR-F | GCAGTAGTTCTACCATGGGATTTC | 236 | NC_007118.7 |
|  | *brsk2b*-gRNA-I4-PCR-R | AGGCTTCTGACTCACTGAGATTTG |  |  |
| RT-qPCR | | | | |
| *brsk2b* | *brsk2b*-E11/12-qPCR-F | TCTCCACTTTTGACGAGGCA | 131 | XM_009303428.2 |
|  | *brsk2b*-E12/13-qPCR-R | TTACTCTGCCCGTGTTGTGC |  |  |
| *brsk2a* | *brsk2a*-E1-qPCR-F | AGCACTACCCCTCATGCCAA | 84 | XM_017354251.2 |
|  | *brsk2a*-E1/2-qPCR-R | AAGCTTTACAAGACCTGTCTGTCCT |  |  |
| *β-actin* | *β-actin*-E2-qPCR-F | CGAGCTGTCTTCCCATCCA | 102 | NM_131031.2 |
|  | *β-actin*-E3-qPCR-R | TCACCAACGTAGCTGTCTTTCTG |  |  |
| *homer1b* | *homer1b*_E1/2-qPCR-F | ATGGATCGAAGGCAATAATAAACAGCA | 243 | NM_001002496.4 |
|  | *homer1b*_E2/3-qPCR-R | GCGATTCCTGAGAGGGCGAAC |  |  |
| *nrgna* | *nrgna*_E1/2-qPCR-F | TGTCGAAACGAAGGATGCAGTCAG | 178 | NM_001302620.1 |
|  | *nrgna*_E2/3-qPCR-R | GCTACTTCGGCTCGCGTTGTTT |  |  |
| *isl1a* | *isl1a*_E2/3-qPCR-F | CTACATCAGGTTATACGGGATCAAATGTG | 280 | NM_130962.1 |
|  | *isl1a*_E3/4-qPCR-R | AATGGGCTCTGCTGCCATTTGTA |  |  |
| *sox2* | *sox2*_E1-qPCR-F | AACGGCACGATTCCCTTATC | 125 | NM_213118.1 |
|  | *sox2*_E1-qPCR-R | TGTGTTCTTTCCTTGAGCTCTATAC |  |  |
| *neurog1* | *neurog1*_E1/2-qPCR-F | CCACCAATAAGGTTATCAACAATGGAGAT | 158 | NM_131041.1 |
|  | *neurog1*_E2-qPCR-R | GCTGGAGACGCAGGTGGTTTTC |  |  |
| *olig1* | *olig1*_E1/2-qPCR-F | GAAAGTTTGAAGAATGCAGGCTGTG | 195 | XM_005167653.4 |
|  | *olig1*_E2-qPCR-R | AGCTCCTGTTGCTCCTCCGAAC |  |  |
| *olig2* | *olig2*_E1/2-qPCR-F | GCTACCGGCAATATCGACATCCT | 185 | NM_178100.1 |
|  | *olig2*_E2-qPCR-R | CTCCGGTGGAGAATCGCTCTG |  |  |
| *sox10* | *sox10*_E2/3-qPCR-F | AGCTGTGGAGACTGCTGAACGAGA | 283 | NM_131875.1 |
|  | *sox10*_E3/4-qPCR-R | TGACTCTGACCTGTAGCGTGAGGG |  |  |
| *mbpa* | *mbpa*_E1/2-qPCR-F | AGAAAGGGAAAGAGACCCCACCAC | 198 | XM_002665561.2 |
|  | *mbpa*_E4-qPCR-R | TCCTGCTTGGAGAAGGGCTCAG |  |  |
| *mpz* | *mpz*_E1/2-qPCR-F | CTTGGGCATAGCCTCTCAGAGCA | 193 | NM_194361.2 |
|  | *mpz*_E2/3-qPCR-R | ACCTCCATAGTGGAAAATTGAGATTGC |  |  |
| *plp1b* | *plp1b*_E1/2-qPCR-F | TCACTGTCATGGCAGACTTTATAAAGTATTTC | 194 | NM_001005586.2 |
|  | *plp1b*_E2/3-qPCR-R | GTACGTCAGGATTATAAAAGTAAGACTTATGCAG |  |  |
