# Supplementary material for: Deleterious Variation in BR Serine/Threonine Kinase 2 Classified a Subtype of Autism
Source: Front Mol Neurosci. 2022 Jun 10;15:904935. doi: 10.3389/fnmol.2022.904935 (PMC9231588; doi:10.3389/fnmol.2022.904935)
Supplement: Supplementary file 3 [file Table_2.docx]

**Supplementary Table 2**

Patient data of *BRSK2* variants and associated phenotypic information.

| Proband | 1 | 2(Hiatt et al., 2019) | 3(Hiatt et al., 2019) | 4(Hiatt et al., 2019) | 5(Hiatt et al., 2019) | 6(Hiatt et al., 2019) | 7(Hiatt et al., 2019) | 8(Hiatt et al., 2019) | 9(Hiatt et al., 2019) | 10(Hiatt et al., 2019) | 11(Feliciano et al., 2019) | 12(Feliciano et al., 2019) | 13(Feliciano et al., 2019) | Overall |
| --- | --- | --- | --- | --- | --- | --- | --- | --- | --- | --- | --- | --- | --- | --- |
| Gender | Male | Male | Male | Female | Male | Male | Male | Male | Male | Male | Male | Male | Male | 12/13, male |
| Age at last evaluation(years) | 4.5 | 5 | 14 | 5.6 | 3.8 | 19 | 4 | 6.8 | 10.9 | 4.6 | 8 | 19 | 5.3 | 8.5±5.5 |
| Gestational age | Full-term birth | Post-term birth, 42 weeks | Full-term birth | Unknown | Unknown | Full-term birth | Full-term birth | Full-term birth | Unknown | Unknown | Unknown | Premature birth, 24weeks | Unknown | —— |
| Variant | c.664C>T  （p. R222X） | c.194G>A  (p.R65Q) | c.273–1G>A | c.530＋1G>A | c.635G>A  (p. G212E) | c.730C>T  (p. Q244X) | c.1281_ 1287+5del12 | c.1395_1396del  (p. S466Qfs*83) | c.1532_1533del  (p. E511Vfs*38) | c.1861C>T  (p.R621C) | p. T547fs | c.951-1G＞A | c.1365-1G＞C | —— |
| Predicted effect | Nonsense | Missense | Splice alteration | Splice alteration | Missense | Nonsense | Frameshift | Frameshift | Frameshift | Missense | Frameshift | Splice alteration | Splice alteration | —— |
| Inheritance | *de novo* | *de novo* | *de novo* | *de novo* | *de novo* | *de novo* | unknown | *de novo* | Not maternal | Unknown | *de novo* | *de novo* | *de novo* | —— |
| Intellectual Disability (YES/ NO) | NO, FSIQ=81 | YES, moderate | YES, severe | YES, mild | YES, moderate | YES, mild to severe, FSIQ=77 at 5 years | Unknown | YES, mild | YES, moderate | YES, moderate | YES | YES, severe, IQ≤25 | Unknown | 10/13 |
| ASD diagnosis | YES, at 19 months | NO | YES | YES, at 5.5 years | YES | YES | YES, at 21 months | YES | YES | YES, borderline | YES | YES | YES | 12/13 |
| ADHD diagnosis | NS | NS | YES | NO | NS | YES, at 18 years | NS | NO | YES | NS | NO | NO | NO | 3/13 |
| Speech delay at first evaluation | YES | YES | YES, with regression | YES | YES, with regression | YES, with regression | YES | YES | YES | YES | YES | YES | YES | 13/13, 3/13 regression |
| Age of speaking (months) | 25 | Unknown | 24 | 18 | Unknown | Unknown | 24 | After 24 | Nonverbal | Unknown | Unknown, single words at evaluation | Nonverbal | Unknown | —— |
| Motor delay at first evaluation | YES | YES | YES | YES, fine motor | NO | YES, fine motor, with regression | YES, gross motor | YES, fine motor | YES, gross motor | YES | YES | YES | NO | 11/13, 1/13 regression |
| Age of walking (months) | 18 | 18 | 17 | 15 | 13 | 12 | 14 | 15 | 24 | 18-20 | Unknown | Unknown | Unknown | —— |
| Morphological features | None reported | Flat nasal bridge, short philtrum, pouting lower lip, full eyelids, high forehead, widely spaced nipples, torticollis | None reported | Upslanting palpebral fissures, large eyes, beaked shaped nose | Downslanting palpebral fissures | Mild  Upslanting palpebral fissures, synophrys, deep-set eyes, short first digits of feet | Close set eyes, transverse palmar crease on the left, supernumerary nipple on the right | Upslanting palpebral fissures, brachycephaly, broad/prominent forehead, narrow nose, long philtrum, broad mouth with thick lower lip and cupid’s bow upper lip, triangular face with pointed chin | Heart-shaped face, down slanting palpebral fissures | Retrognathia, telecanthus, epicanthal folds, upturned ear lobes, oval-shaped face | Unknown | Unknown | Unknown | —— |
| Other comorbidities | Feeding problem, enuresis | Hypermetroia，intermittent honrizontal nystagmus | Sleep disorder, epilepsy at 9 years old | Dysoraxia, mild gait ataxia, tremors | Undescended teste | Disintergration disorder, schizophrenia | Sleep disorder, mild hypotonia | Epilepsy twice | None reported | Mild laryngomalacia and subglottic stenosis, sleep apnea, astigmatism, two episodes of tachycardia | Learning disorder, feeding disorder, speech articulation problem | Feeding problem, vision/hearing, encopresis, enuresis | None reported | —— |

Feliciano, P., Zhou, X., Astrovskaya, I., Turner, T.N., Wang, T., Brueggeman, L., et al. (2019). Exome sequencing of 457 autism families recruited online provides evidence for autism risk genes. *NPJ Genom Med* 4**,** 19. doi: 10.1038/s41525-019-0093-8.

Hiatt, S.M., Thompson, M.L., Prokop, J.W., Lawlor, J.M.J., Gray, D.E., Bebin, E.M., et al. (2019). Deleterious Variation in BRSK2 Associates with a Neurodevelopmental Disorder. *Am J Hum Genet* 104(4)**,** 701-708. doi: 10.1016/j.ajhg.2019.02.002.
